# Supplementary material for: A population model reveals a surprising role of stochastic cell division in epigenetic memory systems
Source: iScience. 2025 Aug 25;28(9):113431. doi: 10.1016/j.isci.2025.113431 (PMC12496179; doi:10.1016/j.isci.2025.113431)
Supplement: Document S1. Figures S1–S9 and Tables S1–S5 [file mmc1.pdf]

iScience, Volume *28*

Supplemental information

A population model reveals a surprising role of stochastic cell division in epigenetic memory systems

Viviane Klingel, Dimitri Graf, Sara Weirich, Albert Jeltsch, and Nicole E. Radde

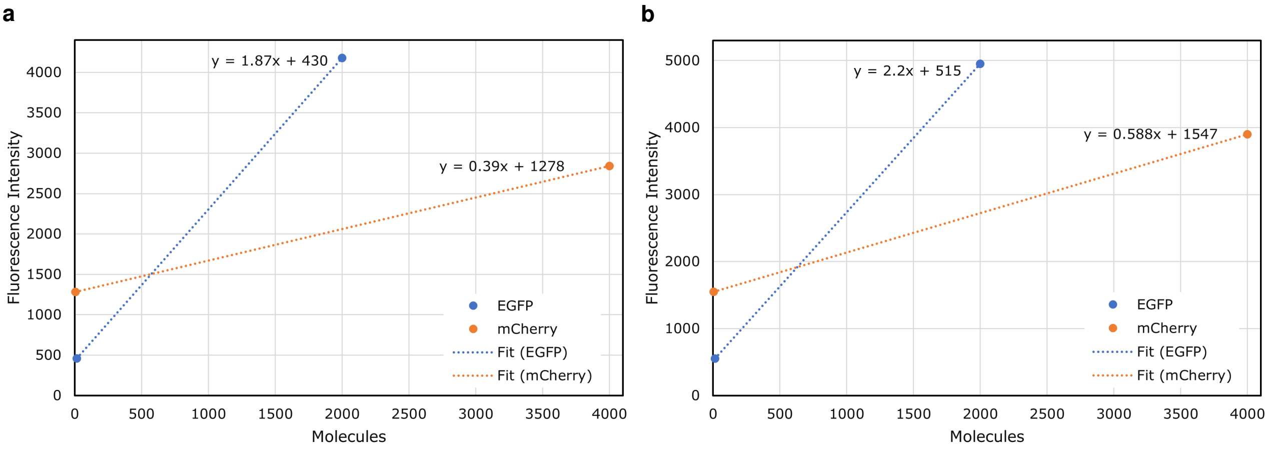

Figure S1: Mapping Fluorescence Intensities to Measured Protein Amounts. Related to Equation 8 and STAR Methods. **a:** Main Experiments. EGFP: $y = 1.87x + 430$, mCherry: $y = 0.39x + 1278$, where x is the number of CcrM molecules per cell and origin, and y the corresponding fluorescence intensity. **b:** Validation Experiments: EGFP: $y = 2.2x + 515$, mCherry: $y = 0.588x + 1547$

Table S1: DNA Methylation Measurements and Ranges. Related to Figure 1 and 4. Measurements of DNA methylation levels from three independent experiments (Rep 1 - 3) over nine days from Graf et al.[1]. The lower and upper limits of the ranges, in which the actual DNA methylation can lie, are given as well. The upper limit is the maximal value of the replicates, while the lower limit is 0.5x the minimal value.

Day	Rep 1	Rep 2	Rep 3	Range Min	Range Max
0	0.038	0.047	0.011	0.005	0.047
1	0.780	0.968	0.972	0.390	0.972
2	0.848	0.745	0.707	0.354	0.848
3	0.511	0.418	0.540	0.209	0.540
4	0.301	0.246	0.301	0.123	0.301
5	0.215	0.244	0.163	0.082	0.244
6	0.131	0.165	0.124	0.062	0.165
7	0.020	0.108	0.101	0.051	0.108
8	0.072	0.064	0.080	0.032	0.080

Table S2: Additional Parameter Sets. Related to Table 1. Parameter values from five representative sets for the two groups each. F1-F5 are sets which correspond to group 1, where quickly dividing cells switch of sooner, while S1-S5 belong to group 2, where slowly dividing cells switch of sooner. The set S1 was used in Figures 4-7 and F1 was used in Figure 6.

	J_{opt}	Kol_{fcm}	LS_{MS}	Parameters							
				a_1	d_1	a_2	d_2	p	k_3	k_4	d_4
S1	26.17	26.13	0.04	-3.704	1.284	-2.310	-1.577	1.478	-0.486	0.297	-1.056
S2	27.03	26.99	0.04	-3.468	1.420	-2.262	-1.170	1.147	-0.604	0.305	-1.016
S3	28.24	28.09	0.15	-3.525	1.359	-2.344	-1.134	1.406	-0.651	0.355	-0.931
S4	26.74	26.7	0.04	-4.079	0.513	-2.065	-1.520	1.700	-0.565	0.310	-1.009
S5	27.57	27.53	0.04	-3.722	1.174	-2.237	-1.150	0.784	-0.505	0.258	-1.077
F1	28.42	28.01	0.41	-3.803	1.311	-1.453	0.699	1.240	-0.556	0.329	-0.968
F2	27.02	26.67	0.35	-4.554	0.151	-1.766	0.182	1.236	-0.639	0.331	-0.949
F3	31.12	30.43	0.69	-4.079	0.941	-1.119	0.906	1.689	-0.664	0.306	-1.000
F4	30.93	29.3	1.63	-4.065	1.009	-1.471	0.667	1.469	-0.683	0.333	-0.963
F5	34.47	32.72	1.75	-3.750	0.422	-1.058	0.175	1.478	-0.627	0.448	-0.869

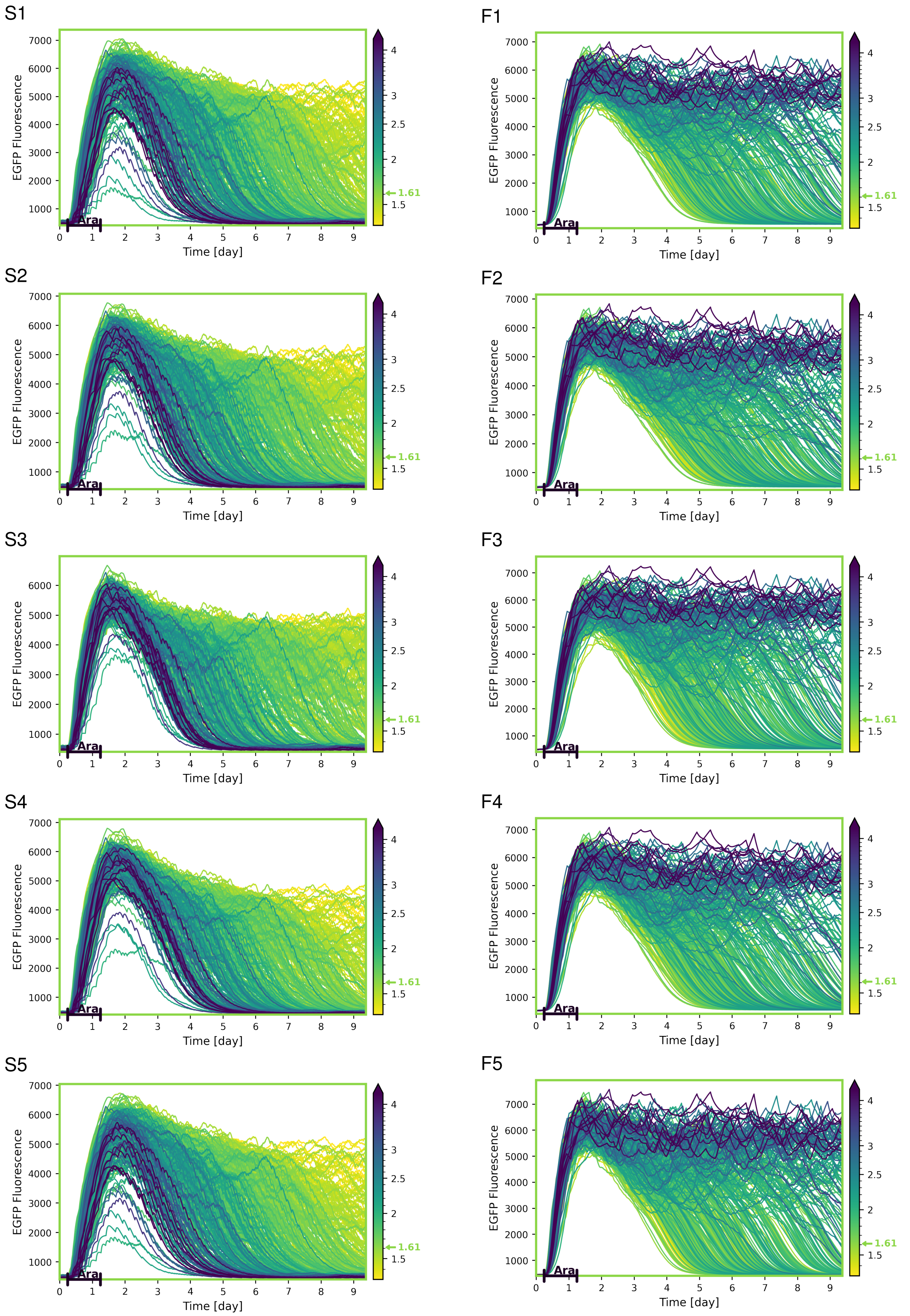

Figure S2: Simulation Additional Parameter Sets. Simulation of the five slow-off parameter sets S1-S5 and the five fast-off parameter sets F1-F5. Related to Table 1 and Figure 6.

Table S3: Objective Function Values S1. Related to STAR Methods. Objective function values for the parameter set S1, showing slow-off behavior. The model was simulated 50 times for each of the three sets (WT) of randomly sampled cell-cycle duration times (T_{div}) and plasmid numbers (Z_P).

WT Set	J_{opt}									
	25.03	25.26	28.1	28.29	28.22	29.33	26.45	28.13	27.4	25.37
	26.81	24.01	26.93	27.18	26.47	24.54	26.98	27.18	25.31	26.95
1	26.24	26.91	26.41	26.57	27.43	28	27.48	27.49	25.37	25.43
	28.51	27.9	26.64	26.41	28.48	26.35	27.04	28.61	26.34	26.12
	28.16	27.63	25.07	27.35	26.99	27.15	27.77	26.45	26.41	25.63
	26.39	27.65	26.64	27.48	27.04	27.11	26.59	26.08	26.72	27.31
	25.55	28.43	28.04	27.49	25.15	25.89	26.42	26.16	26.19	26.93
2	27.37	26.35	28.2	26.76	26.29	27.37	27.6	27.56	28.39	24.74
	28.15	26.08	26.21	26.46	26.13	26.1	27.79	25.43	27.39	25.75
	27.68	23.73	26.25	26.46	27.16	26.85	28.84	25.72	26.78	25.24
	26.58	27.49	27.42	26.37	26.12	26	26.14	27.74	26.65	26.42
	26.71	27.3	25.44	26.5	29.62	27.39	28.43	28.82	27.26	27.92
3	28.87	29	25.44	28.2	27.72	27.47	25.04	28.1	27.04	27.76
	27.48	26.87	26.37	28.02	25.95	27.12	27	27.34	26.9	27.46
	24.98	27.7	27.56	27.43	27.57	25.65	26.42	27.07	26.76	24.56

Table S4: Objective Function Values F1. Related to STAR Methods. Objective function values for the parameter set F1, showing fast-off behavior. The model was simulated 50 times for each of the three sets (WT) of randomly sampled cell-cycle duration times (T_{div}) and plasmid numbers (Z_P).

WT Set	J_{opt}									
	30.12	31.69	30.61	29.59	30.15	29.27	28.88	29.18	30.51	29.53
	27.5	30.75	29.03	29.46	30.2	29.82	29.19	29.42	29.94	30.23
1	32.03	30.6	29.25	30.54	29.84	28	29.55	31.65	31.01	28.11
	29.58	28.89	28.6	31.63	29.95	29.6	30.34	30.17	31.14	29.79
	29.19	28.42	32.57	30.32	29.28	29.72	31.78	29.34	29.7	28.89
	29.46	29.04	28.99	31.24	29.12	31.88	32.63	33.1	31.13	31.13
	32.47	31.48	30.62	31.5	29.07	32.06	31.15	32.48	32.86	32.69
2	31.14	30.88	32.8	32.22	31.07	32.46	31.01	28.48	30.06	29.93
	30.24	30.06	30.14	31.15	31.22	31.79	31.62	30.78	31.06	27.64
	31.61	31.44	29.86	31.86	31.77	30.81	31.93	30.76	32.68	29.22
	31.68	32.89	30.89	31.72	30.98	30.48	31.51	31.85	30.6	32.51
	31.01	31.16	31.04	30.83	32.46	29.39	32.76	32.05	31.59	29.18
3	31.48	29.72	32.13	32.05	29.51	31.99	28.33	31.36	29.55	29.98
	30.49	29.9	31.55	31.24	32.8	31.26	30.66	31.41	29.28	31.2
	32.37	29.3	33.55	30.41	30.34	31.4	31.01	32.62	31.88	30.34

Table S5: Comparison of Objective Function Values. Related to STAR Methods. The two parameter sets S1 (Slow-Off) and F1 (Fast-Off) were simulated 50 times for each of the three sets of randomly sampled cell-cycle duration times (T_{div}) and plasmid numbers (Z_P). The table shows the mean, standard deviation, minimum, and maximum over the objective function values of those 50 simulations (as given in Table S3 and S4).

	Div-Set	Mean	SD	Min	Max
Slow-Off	1	26.85	1.15	24.01	29.33
	2	26.72	1.01	23.73	28.84
	3	27.06	1.06	24.56	29.62
Fast-Off	1	29.89	1.06	27.50	32.57
	2	31.04	1.26	27.64	33.10
	3	31.11	1.13	28.33	33.55

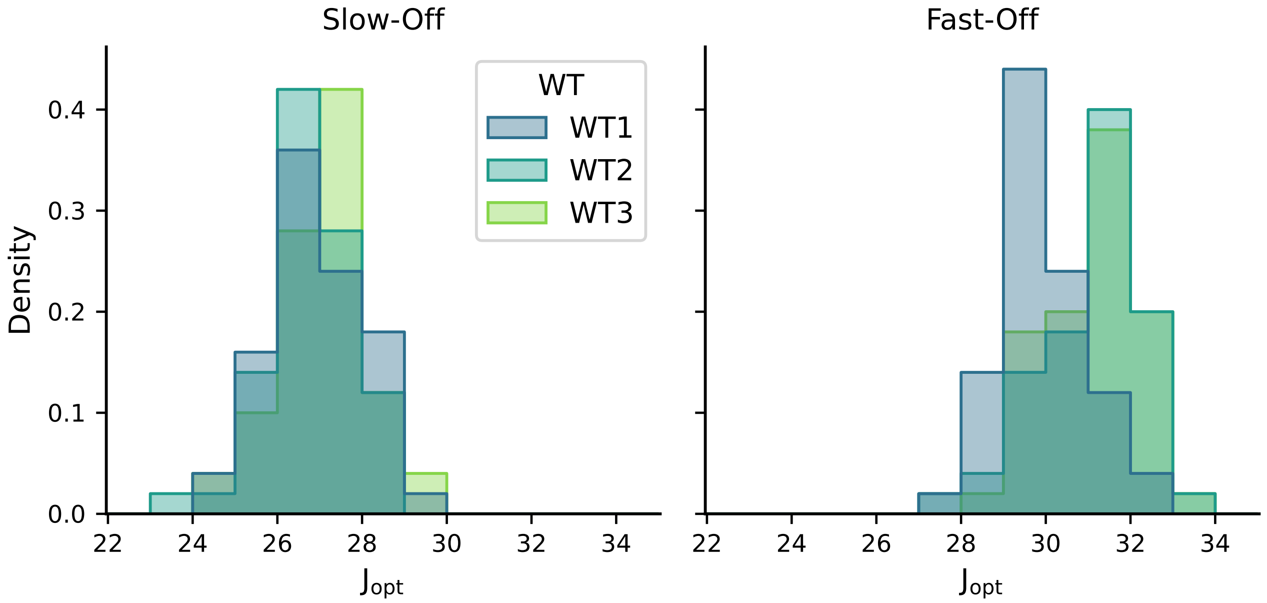

Figure S3: Histograms of Objective Function Values. Related to STAR Methods. Comparison of objective function values for the two parameter set S1 (Slow-Off) and F1 (Fast-Off).

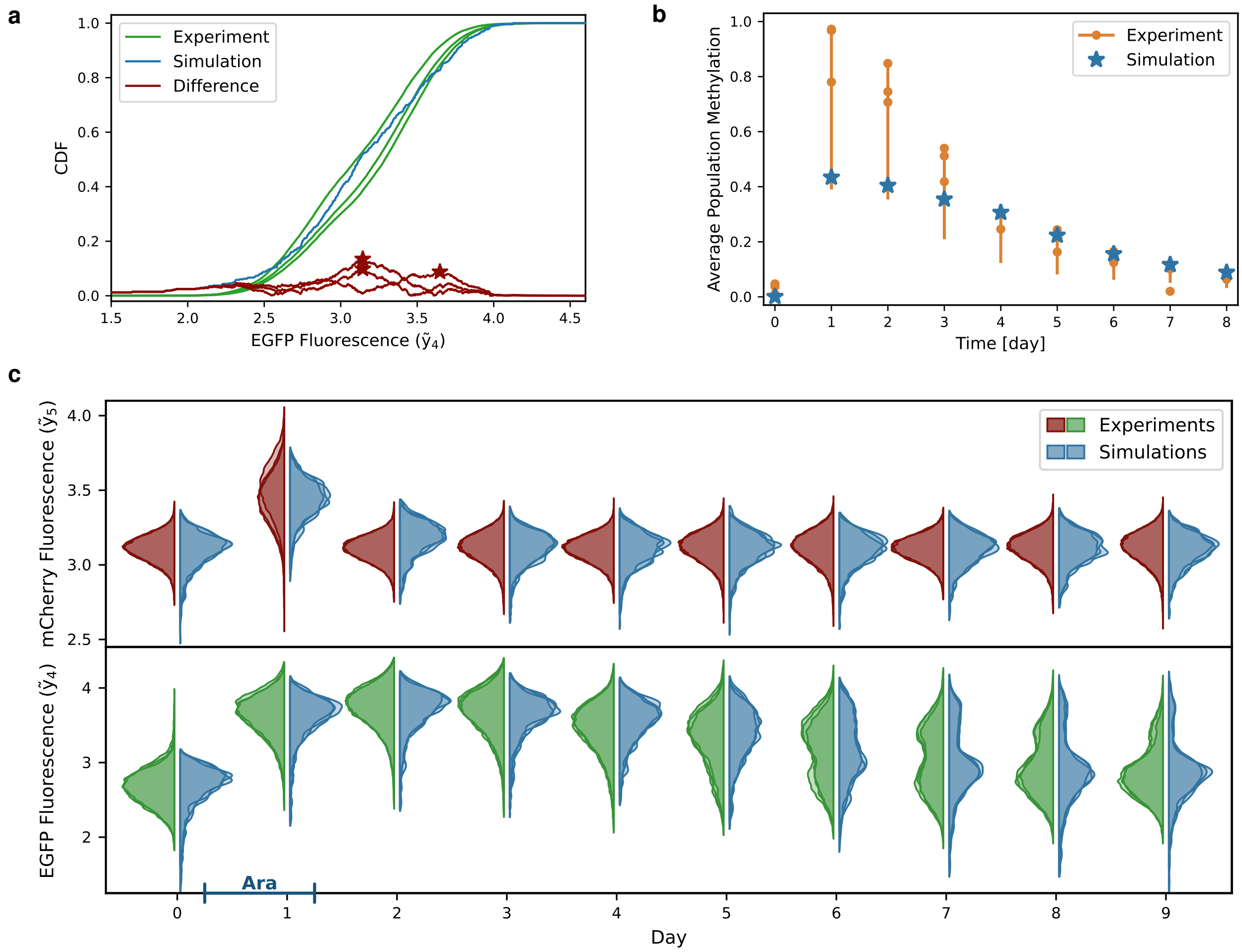

Figure S4: Optimization and Model Fit for Parameter Set F1. Related to Figure 4 and 6. Experimental data from Graf et al.[1] and simulations from one representative parameter set, which shows increased OFF-switching for quickly dividing cells. **a:** Cumulative densities (CDF) for data and simulation of EGFP fluorescence. The red stars indicate the Kolmogorov metric as the largest difference between the CDFs of each of the data replicates and the simulation, shown for one of the ten measured days. **b:** Measured and simulated DNA methylation levels. **c:** Violin plots of the measured and simulated densities of mCherry and EGFP over ten days. The fluorescence intensities in a and c are shown in a logarithmic scale.

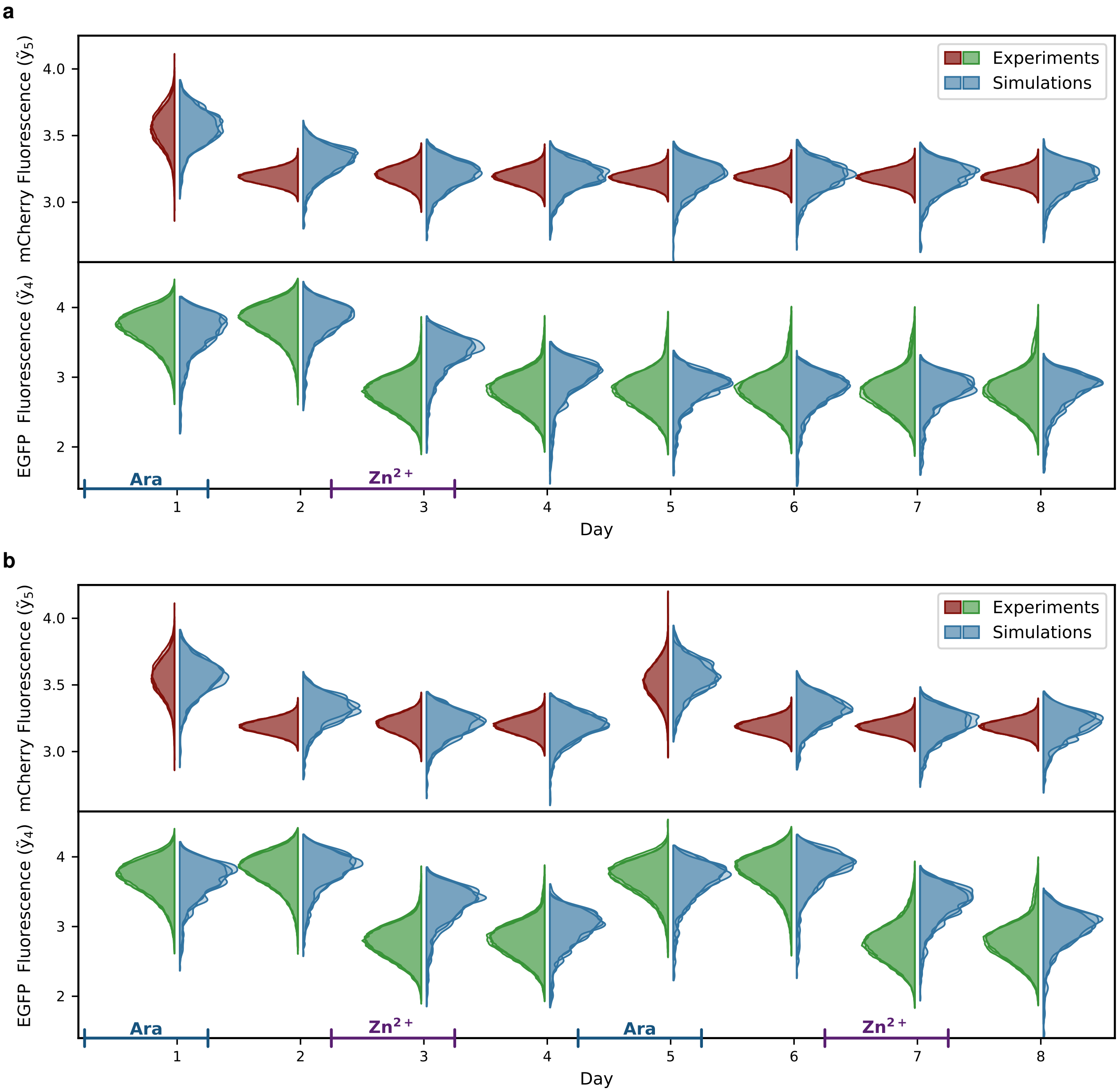

Figure S5: Simulation and Data of Further Validation Experiments. Related to Figure 5. Experimental data from Graf et al.[1] and simulation for the ON-OFF (**a**) and ON-OFF-ON-OFF (**b**) validation experiments.

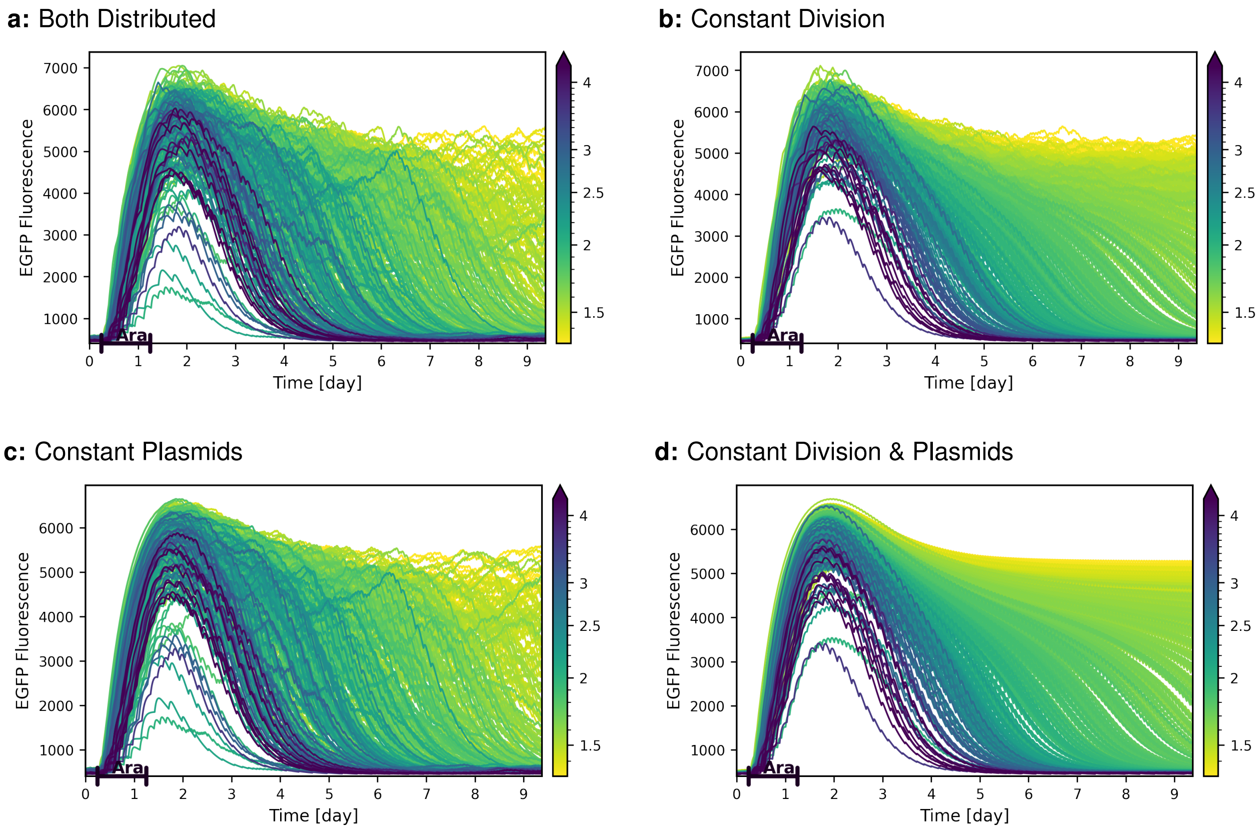

Figure S6: Sources of Heterogeneity S1. Related to Section *"Hybrid Model Captures Slow OFF-Drift"*. Effect of different sources of heterogeneity on the simulation results of parameter set S1.

a: Full model with both randomly sampled inter-division times (T_{div}) and plasmid numbers (Z_P) for every cell and division.

b: Randomly sampled plasmid numbers for every division, but a constant inter-division time for each cell, corresponding to the average over all inter-division times of that cell during the entire simulation period.

c: Randomly sampled inter-division times for every division, but constant plasmid numbers for each cell, corresponding to the average over all plasmid numbers of that cell during the entire simulation period.

d: Both constant inter-division times and constant plasmid numbers for each cell, corresponding to the averages over all inter-division times or plasmid numbers of that cell, respectively, during the entire simulation period.

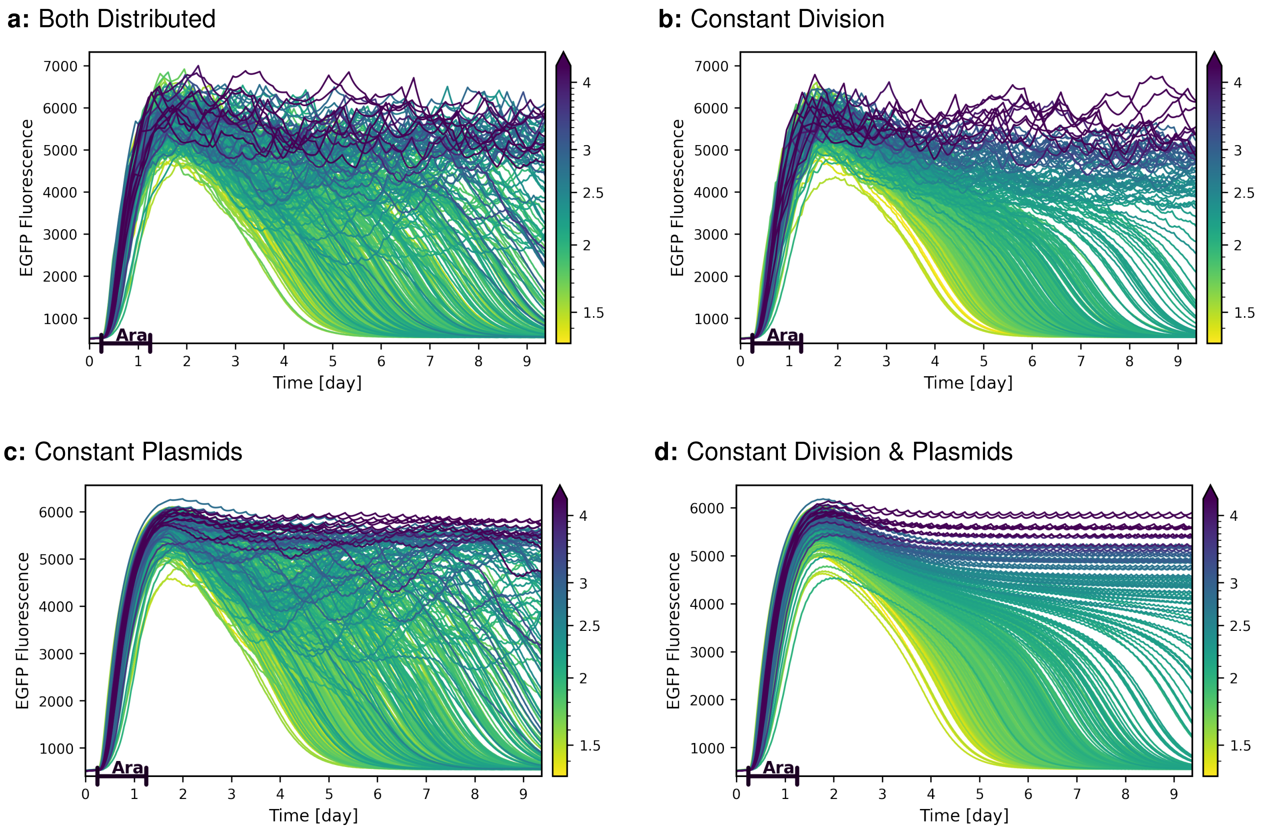

Figure S7: Sources of Heterogeneity F1. Related to Section *"Hybrid Model Captures Slow OFF-Drift"*. Effect of different sources of heterogeneity on the simulation results of parameter set F1.

a: Full model with both randomly sampled inter-division times (T_{div}) and plasmid numbers (Z_P) for every cell and division.

b: Randomly sampled plasmid numbers for every division, but a constant inter-division time for each cell, corresponding to the average over all inter-division times of that cell during the entire simulation period.

c: Randomly sampled inter-division times for every division, but constant plasmid numbers for each cell, corresponding to the average over all plasmid numbers of that cell during the entire simulation period.

d: Both constant inter-division times and constant plasmid numbers for each cell, corresponding to the averages over all inter-division times or plasmid numbers of that cell, respectively, during the entire simulation period.

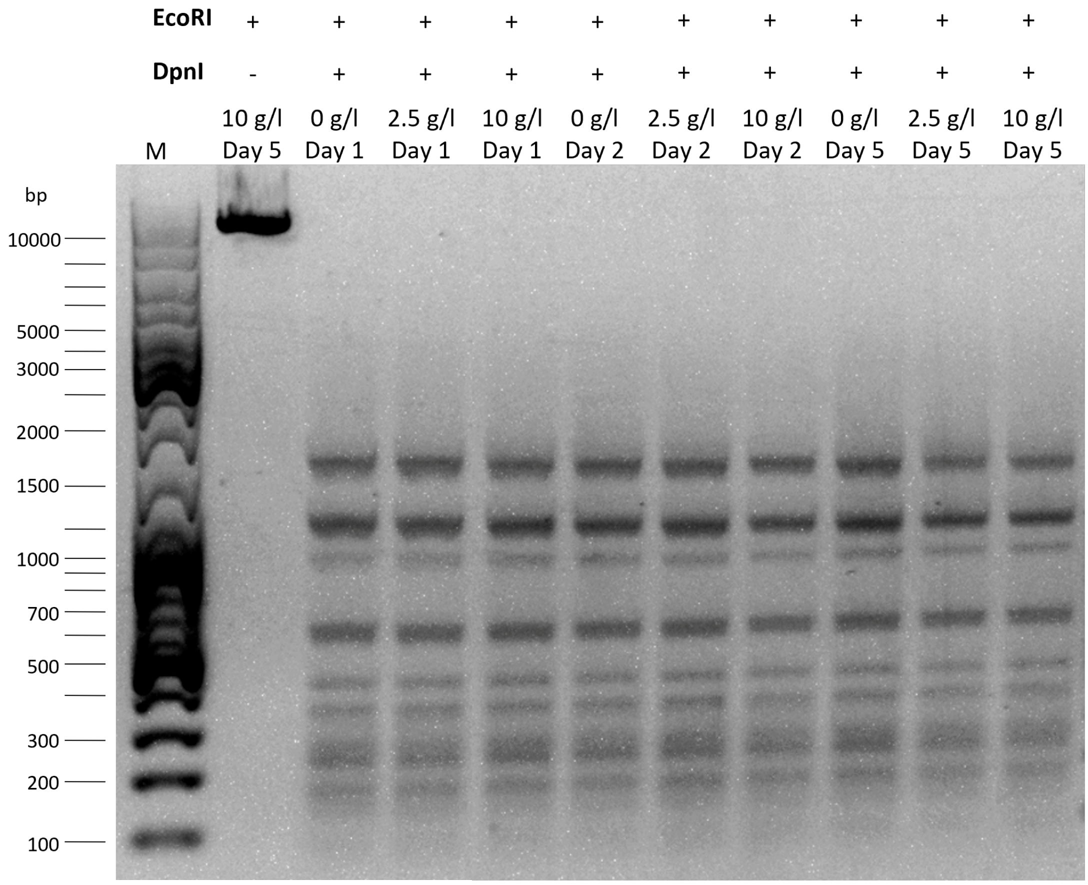

Figure S8: Analysis of Global DNA Methylation Levels by Restriction Digestion Analysis. Related to Section *"New Experiments Surprisingly Favor Scenario 2"*. Agarose gel electrophoresis performed on genomic DNA digested with the methylation sensitive DpnI restriction enzyme. 0, 2.5 and 10 g/L indicated the different peptone concentration in the cultivation medium. The purified plasmids were linearized with EcoRI and digested with the DpnI restriction enzyme, which cuts the sequence GATC only when the adenine was methylated before by the DAM methyltransferase. No change in the methylation pattern could be observed between all samples, which means that lowering the peptone amount in the cultivation medium did not reduce the global DNA methylation in the cell. As a control resembling the unmethylated plasmid, one sample was digested solely with EcoRI. M refers to the molecular weight marker.

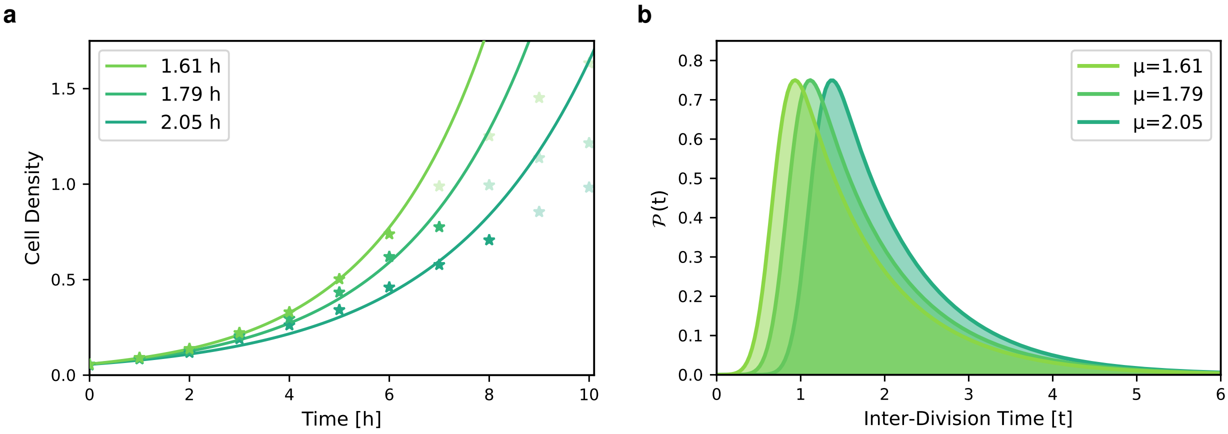

Figure S9: Measurement and Fit of Cell Growth Rates. Related to Figure 7. Cell growth curves under different peptone concentrations were measured and used to determine the cell divisions times. These were translated into exponentially modified normal distributions by shifting the original distribution to higher division times. **a:** Exponential cell growth fits used to extract cell division times. Only measurements during the exponential growth phase were used for the fit, markers with reduced opacity indicate when cell growth starts to enter the stationary phase. The three curves correspond to three conditions with differing peptone concentrations (1.61 h: 10 g/L, 1.79 h: 2.5 g/L, 2.05 h: 0 g/L). **b:** Translation of measurements into model densities for cell division times.

References

1. Graf, D., Laistner, L., Klingel, V., Radde, N., Weirich, S., and Jeltsch, A. (2023). Reversible switching and stability of the epigenetic memory system in bacteria. FEBS J *290*, pp. 2115–2126. doi:10.1111/febs.16690.
